# *Satureja khuzestanica* Essential Oil against Quorum Sensing of *Pseudomonas aeroginosa* Using RT-PCR

**Published:** 2019-07

**Authors:** Parya BABAN ZADEH, Azad KHALEDI, Davoud ESMAEILI

**Affiliations:** 1. Department of Microbiology, Faculty of Pharmaceutical Sciences, North Tehran Branch, Islamic Azad University, Tehran, Iran; 2. Department of Microbiology and Immunology, Faculty of Medicine, Kashan University of Medical Sciences, Kashan, Iran; 3. Department of Microbiology and Applied Microbiology Research Center, Systems Biology and Poisonings Institute, Baqiyatallah University of Medical Sciences, Tehran, Iran; 4. Applied Virology Research Center, Baqiyatallah University of Medical Sciences, Tehran, Iran

## Dear Editor-in-Chief

*Pseudomonas aeruginosa* is considered as an important opportunist nosocomial pathogen, which in fact is highly resistant to most common antibiotics. For this reason, the treatment of infections resulted from this pathogen (*P.aeruginosa*) creates many problems (1,2). Different virulence factors (VFs) are produced by this bacterium which most of these extracellular VFs are synthesized under regulatory systems of Quorum sensing (3). Quorum sensing makes the connection between bacteria. This allows intra-isolates as well as inter-isolates connections among bacteria. There are two systems of Quorum sensing, such as rhl and las, in *P. aeruginosa*. These are known systems used to control the expression of some virulence genes (4).

Carvacrol is considered as one of the most effective compounds of *Satureja khuzestanica*, which affects membrane, protein, and genes. This can affect the electron transport chain, and even can be used as food (5,6). Therefore, this study aimed to assess the inhibitory effects of carvacrol on the expression of quorum sensing of *P. aeruginosa* using RT-PCR.

The shoot parts of *S. khuzestanica* were collected over the third months of spring (2013) from Lorestan Province, Iran and its essence were extracted and analyzed to evaluate its antimicrobial activity by Microdilution method. Minimum Inhibitory Concentration (MIC) essence was performed in accordance with the Clinical & Laboratory Standards Institute (CLSI) 2013 guidelines. To analyze the expression of quorum sensing, mRNA of the *P. aeruginosa* isolates (recovered from Motahari Hospital, Tehran) was extracted and cDNA synthesized, and RT-PCR reaction performed using kit (Cinnagen Company, Iran). Combination of materials for the reaction was as follows; Primers (Forward, Reverse) and DNA sample: 1μL; Master mix: 12.5; distilled water (DDW): 9.5 μL in final volume: 25 μL. The program of PCR reaction was; Hot start 3 min at 95 °C for 1 cycle, denaturation 30 sec at 95 °C, then, 35 cycles at 54 °C for 30 sec and at 72 °C for 1 min; finally, at 72 °C for 10 min.

Finally, results analyzed using SPSS software (ver. 22, Chicago, II, USA) and non-parametric statistical test (Kruskal Wallis) and *P*-value less than *P*<0.05 was considered significant.

The results of susceptibility testing showed that all 5 isolates were resistant against Gentamycin, Tobramycin, and Ciprofloxacin and all of those were susceptible to Polymyxin B and 50% of isolates were resistant to Amikacin. The essence has a favorable anti-bacterial effect.

**Fig. 1:**
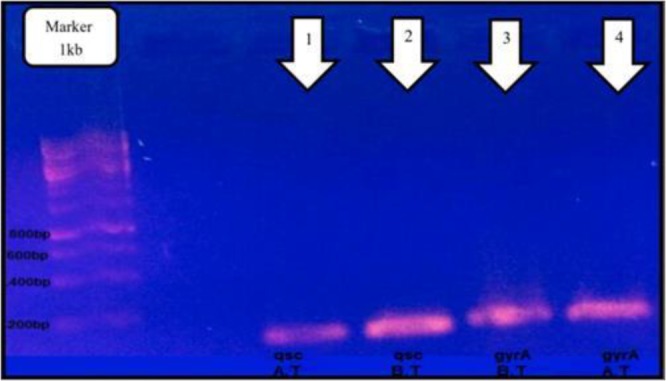
RT-PCR results for expression of ‘(QS)’ and *gyrA* genes after treatment with *Satureja Khuzestanica*, B.T: Before treatment, A.T: After treatment

The amount of MIC, for all 5 isolates, based on Microdilution method and according to CLSI was 31% (3.1 mg/mL). Moreover, the expression of ‘(QS)’ gene was decreased after treatment with S.Khuzestanica, whereas, the expression of the *gyrA* gene had remained constant throughout the study (Fig. 1).

The bacteria can communicate with each other through the phenomenon of Quorum sensing (QS) and adapt themselves to genomic expression patterns in case of environmental changes. Moreover today, with restrictive QS, anti-bacterial drugs produce (7,8) in the control of infections in medical, agricultural and industrial applications (9). We believe that some of the antimicrobial properties of HSMP may be contributed by the QS inhibiting phytochemicals present in it.

In this study, the RT-PCR technique was utilized to survey the effect of *S. khuzestanica* essence on the expression of Quorum sensing pathogenic gene. Herbal essence is one of the potential sources of anti-bacterial compounds and useful in making antimicrobial compounds. Therefore, the present study introduced this compound as a medicine and health supplement to inhibit *P.aeruginosa*. *S. khuzestanica* has an inhibitory effect on the expression of quorum sensing of *P. aeruginosa*.

The essence of *S. khuzestanica* reduced the expression of quorum sensing during 24 h. Therefore, this essence is effective against *P. aeruginosa* due to inhibition of gene expression of *P. aeruginosa* and anti-bacterial effect.
